# Comparison of Different Pretreatment Processes Envisaging the Potential Use of Food Waste as Microalgae Substrate

**DOI:** 10.3390/foods13071018

**Published:** 2024-03-26

**Authors:** Fabiana Marques, Francisco Pereira, Luís Machado, Joana T. Martins, Ricardo N. Pereira, Monya M. Costa, Zlatina Genisheva, Hugo Pereira, António A. Vicente, José A. Teixeira, Pedro Geada

**Affiliations:** 1 CEB—Centre of Biological Engineering, University of Minho, 4710-057 Braga, Portugal; fabiana.almeida.marques@hotmail.com (F.M.); franciscopereira@ceb.uminho.pt (F.P.); luismachado@ceb.uminho.pt (L.M.); joanamartins@ceb.uminho.pt (J.T.M.); rpereira@ceb.uminho.pt (R.N.P.); jateixeira@deb.uminho.pt (J.A.T.); pedrogeada@ceb.uminho.pt (P.G.); 2 LABBELS—Associate Laboratory, 4710-057 Braga/Guimarães, Portugal; 3 GreenCoLab—Associação Oceano Verde, Universidade do Algarve, Campus de Gambelas, 8005-139 Faro, Portugal; monyacosta@greencolab.com (M.M.C.); hugopereira@greencolab.com (H.P.); 4 CVR—Centre of Wastes Valorization, 4800-058 Guimarães, Portugal; zlatina@cvresiduos.pt

**Keywords:** waste valorization, autoclaving, ohmic heating, circular economy, heterotrophy

## Abstract

A significant fraction of the food produced worldwide is currently lost or wasted throughout the supply chain, squandering natural and economic resources. Food waste valorization will be an important necessity in the coming years. This work investigates the ability of food waste to serve as a viable nutritional substrate for the heterotrophic growth of *Chlorella vulgaris*. The impact of different pretreatments on the elemental composition and microbial contamination of seven retail food waste mixtures was evaluated. Among the pretreatment methods applied to the food waste formulations, autoclaving was able to eliminate all microbial contamination and increase the availability of reducing sugars by 30%. Ohmic heating was also able to eliminate most of the contaminations in the food wastes in shorter time periods than autoclave. However, it has reduced the availability of reducing sugars, making it less preferable for microalgae heterotrophic cultivation. The direct utilization of food waste containing essential nutrients from fruits, vegetables, dairy and bakery products, and meat on the heterotrophic growth of microalgae allowed a biomass concentration of 2.2 × 10^8^ cells·mL^−1^, being the culture able to consume more than 42% of the reducing sugars present in the substrate, thus demonstrating the economic and environmental potential of these wastes.

## 1. Introduction

Food waste and food loss have different definitions, according to the Food and Agriculture Organization of the United Nations (FAO). Food waste is referred to as “the discarding of food products suitable for human consumption”. On the other hand, food loss is used to refer to the “reduction in quantity and quality of food”. Since food waste and loss can occur at every step of the food supply chain (FSC), its specific composition varies according to its origin and intrinsic characteristics [1]. It mostly comprises organic matter composed of 41–62% carbohydrates, 13–30% lipids, and 15–25% proteins [2]. In addition to the general composition, food wastage can also be divided according to the type of food wasted. In 2011, a study was conducted to define the quantity of food wastage generated in the European Union (EU) and concluded that 24% and 22% belong to vegetables and fruits, respectively, representing the main waste streams. These categories were then followed by cereals, meat, crops, fish, and eggs, with 12, 11, 10, 3, and 2%, respectively. Considering the amount of wastage, the demand for food is expected to rise by 50% in 2050 as a consequence of an increase in the human population, which, ultimately, will lead to an escalation in food wastage throughout the FSC and excessive use of limited natural resources [3]. This fact will raise environmental, economic, and social problems associated with the incineration and disposal—mainly in landfills and open disposal sites—of biowaste, leading to an undesirable increase in the carbon footprint as a consequence of the emission of significant quantities of greenhouse gases (GHG) into the atmosphere [4,5,6]. In addition, leachates from landfills can give rise to groundwater contamination, subsequently causing the pollution of adjacent water bodies [6,7].

To promote a circular economy and diminish the use of these non-environmentally friendly approaches, it is important to select different strategies to manage this type of biowaste, such as composting and anaerobic digestion [8]. Both strategies allow food waste valorization into compost/organic fertilizers and biofuels, respectively [6]. However, food waste processing generally has high setup and infrastructure costs, along with high energy consumption [9]. Another challenging aspect of these treatments is the variability in terms of the quality and composition of the food waste, which can lead to reduced efficiency in producing various end products [8]. While composting may have lower initial costs compared with some counterparts, it can cause additional issues, such as unpleasant odors and the proliferation of harmful bacteria [10]. Furthermore, the large scale required for the process and inadequate regulation of the composting process can lead to incomplete decomposition, contributing to GHG emissions [11].

In this sense, food waste valorization using bio-based strategies, such as microalgae cultivation, presents significant potential as a sustainable alternative [12,13,14]. As stated above, food waste can be a nutrient-rich substrate that can represent a low-cost source of sugars, ammonium, and phosphate, known as major nutrients for cell development and microalgae growth [15,16]. Microalgae are an immense group of unicellular photosynthetic microorganisms that, due to their complete nutritional constitution (e.g., rich-protein composition, production of polyunsaturated fatty acids (PUFAs) and pigments), have a vast interest in several biotechnological sectors [17]. Typically, microalgae thrive through photoautotrophic growth, converting solar energy to chemical energy by carbon fixation [18]. However, certain challenges, such as low yields and intermittent light availability, might jeopardize this demanding technology both economically and technically [19]. In this context, an alternative approach emerges in the form of heterotrophic growth, offering a promising solution to these issues. Supplementing microorganisms with organic carbon sources, such as glucose, makes it possible to achieve higher and more consistent yields. Unfortunately, the cost of organic carbon sources can account for up to 80% of the production expenses [20]. Thus, utilizing nutrients recovered from food waste can prove to be economically advantageous in overcoming this situation, promoting a circular economy.

This work aims to characterize different retail food waste (FW) formulations and analyze their potential to support microalgae growth. Therefore, six food categories—fruits, vegetables, dairy, bakery, meat, and fish—from a retail store were used to produce different mixtures of food waste. Additionally, three pretreatment processes—conventional heating, ohmic heating, and autoclaving—were tested to assess their impact on the organic carbon content and efficiency in controlling microbial contamination for subsequent application in the heterotrophic growth of microalgae *Chlorella vulgaris*.

## 2. Materials and Methods

### 2.1. Retail Food Waste

The seven FW formulations used for the development of this work were kindly provided by MC retailer (Continente, Sonae, Matosinhos, Portugal), being the mixtures composed of several food categories—fruits, vegetables, dairy, bakery, fish, and meat—according to Table 1.

### 2.2. Food Waste Media Preparation

The media were obtained after overnight (14 h) solubilization of the FW formulations (Section 2.1) at 250 g·L^−1^, using deionized water as solvent. The process was performed using 1 L Schott flasks with a final volume of 900 mL and occurred at room temperature under constant agitation (600 rpm) using a magnetic plate stirrer (VWR Advanced VMS-C7, Radnor, PA, USA). Afterward, the solution underwent a sedimentation process for 24 h using a decantation funnel, where the liquid phase was separated for further analysis.

### 2.3. Pretreatments

To cultivate microalgae using the collected liquid mixtures from the FW (Section 2.2), it is crucial to identify the most effective sterilization technique and its influence on the mixtures’ composition. Thus, three pretreatment processes—conventional heating, ohmic heating, and autoclaving—were applied in triplicate to assess their impact on the FW media.

#### 2.3.1. Conventional Heating

An adaptation of Pereira et al. [21] was performed to apply the conventional heating (COV) pretreatment in the FW samples (Section 2.2). The samples were heated in a cylindrical water-jacketed glass reactor connected to a temperature-controlled circulatory water bath (Julabo HE-4, Seelbach/Germany). The temperature was set to reach a maximum of 95 °C and maintained at this temperature for 30 s. The temperature of the sample was measured in real-time using a type-K thermocouple U (SB-9161, National Instruments Corporation, Austin, TX, USA) placed at the center of the reactor.

#### 2.3.2. Autoclaving

The autoclaving (AT) process was performed using an autoclave (A.J. Costa, LDa. model Uniclave 77, Cacém, Portugal), where the temperature was set at 121 °C for 40 min.

#### 2.3.3. Ohmic Heating

Ohmic heating (OH) experiments were adapted from Pereira et al. [21]. The reactor used in this protocol was the same as described in Section 2.3.1 but was equipped with two stainless steel electrodes on each side. To ensure proper insulation, polytetrafluoroethylene (PTFE) was used to isolate the edges of those electrodes. For the creation of a sinusoidal electric wave with a small peak voltage, it was used a digital function generator (Agilent 33220A, Penang, Malaysia) with frequency and voltage ranges of 1 Hz to 25 MHz and 1 V to 10 V, respectively. Wave amplification was performed using an amplifier (4505 Precision Power Amplifier from Miko-Kings Instruments Ltd. in Hong Kong, China). The temperature was monitored and adjusted as mentioned in Section 2.3.1, being controlled by applying a constant electric field of 12.5 V.cm^−1^ until the desired temperature (95 °C) was attained. The frequency applied in every treatment was established at 20 kHz.

### 2.4. Nutrients Quantification/FW Characterization

#### 2.4.1. Elemental Characterization

The elemental characterization of each FW formulation was performed using a Vario el III (Vario EL, Elementar Analyser System, Langenselbold, Germany) and using an Agilent Technologies 4200 microwave plasma-atomic emission spectroscopy (Agilent 4200 MP-AES, Santa Clara, CA, USA). The percentage of carbon, nitrogen, and hydrogen, as well as the amount of calcium, iron, potassium, magnesium, sodium, and phosphorus, were determined in all FW formulations (Section 2.2).

#### 2.4.2. Carbon Quantification

##### Reducing Sugars

A modified version of the Bernfeld [22] protocol (dinitrosalicylic acid—DNS—method) was performed for the quantification of reduced sugars. In terms of reducing sugars, a calibration curve was determined using a D-Glucose solution with a concentration ranging between 0 and 2.5 g·L^−1^ to estimate the amount of carbon present in the FW formulations. DNS reagent, containing 10 and 300 g·L^−1^ of 3,5-dinitrosalicylic acid and potassium sodium tartrate, respectively, was applied, in equal volume, to the solubilized by-product samples. After addition, the mixtures containing the reagent were incubated at 100 °C for 5 min. Afterward, the mixtures were cooled by the addition of 5 mL of deionized water, and the absorbance (540 nm) was read in 96-well plates using a SynergyTM HT Multi-detection Microplate Reader (Bio-Tek Instruments, Inc., Winooski, VT, USA).

##### High-Performance Liquid Chromatography

Glucose concentrations were determined using a high-performance liquid chromatography (HPLC) methodology similar to Genisheva et al. [23]. The HPLC methodology was performed using a Shimadzu LC 2060C chromatograph set with a refractive index detector (Shimadzu Corporation, Kyoto, Japan) and a BioRad Aminex HPX—87H (300 mm × 7.8 mm) column operated at 60 °C. For the eluent, a 5 mmol·L^−1^ H_2_SO_4_ aqueous solution was used (filtered with 0.22 µM PES membrane (VWR International, LLC, Radnor, PA, USA)) and degassed with a water vacuum pump (VELP SCIENTIFICA, Usmate Velate, Italy), and injected in the column at a constant flow rate of 0.6 mL·min^−1^. A standard calibration of glucose was performed under the same conditions. To quantify the glucose present in the pretreated FW samples (Section 2.3), the retention times of the peaks obtained were compared with the standard calibration. The quantifications were performed in triplicate, and all samples were filtered with a 0.22 µm nylon filter (Nantong FilterBio Membrane Co., Ltd., Nantong, China).

### 2.5. Chemical Oxygen Demand

The chemical oxygen demand (COD) in the FW samples (Section 2.2) was determined spectrophotometrically using the cuvette test kit LCK 514 (Hach-Lange GmbH, Düsseldorf, Germany) with a range of detention between 100 and 2000 mg·L^−1^, according to the manufacturer’s instructions.

### 2.6. Microbiological Analyses

Microbiological analyses were conducted to determine the effectiveness of each pretreatment on microbial contamination of the FWs. Liquid extracts were subjected to different pretreatments (Section 2.3), and the samples (non-treated and treated) were diluted 10-fold using buffered peptone water (HiMedia Laboratories, Thane, India) under sterile conditions using a laminar air-flow chamber (OPTIMALE 12, ADS Laminaire, Aulnay sous Bois, France). After this process, the different solutions were poured into Plate Count Agar (PCA, VWR Chemicals; Leuven, Belgium) (performed in triplicate) and incubated (WTB Binder, Tuttlingen, Germany) at 30 °C for 7 days. The efficiency of each treatment was evaluated by visually monitoring microbial growth on each plate.

### 2.7. Heterotrophic Cultivation

#### 2.7.1. Culture Maintenance

The microalga strain used was *Chlorella vulgaris* 0002 CA, a freshwater species kindly provided by Allmicroalgae—Natural Products, S.A. (Pataias, Portugal). The cultures were maintained in T-Flasks containing a heterotrophic medium (FERM_MB), previously developed by the company, composed of 4 solutions: macronutrients (MB), micronutrients (TM), vitamins (VIT), and glucose 50% *w*/*v* (as carbon source) [24].

#### 2.7.2. Growth Assay

For the growth assays, all material and media used were autoclaved at 121 °C for 40 min. The experiments were performed using 250 mL Erlenmeyer flasks covered with aluminum foil and a cotton wool plug on top, containing a final volume of 100 mL. *C. vulgaris* was inoculated with an initial optical density (750 nm) of 0.1 in the FW media (*2* and *4*), with a pH level between 6.9 and 7.1. Growth was conducted in a temperature-controlled orbital shaker (IKA KS 4000i, Staufen, Germany), set at 30 °C and 200 rpm. To monitor the growth and pH, a sample (700 µL) was collected at the beginning of the assay, 24 h after, and then two samples every day. The pH was measured using a pH meter (Hanna HI 2210, Padova, Italy) and adjusted with 1 M HCl or 5 M NaOH whenever necessary. On the other hand, growth kinetics was monitored through cell counting using a Neubauer hemacytometer. In the stationary phase, the growth assay was stopped, and the cultures were centrifuged at 6000 rpm for 10 min (ThermoFisher Scientific, Dreieich, Germany) with the supernatant used to evaluate the consumption of reducing sugars (as mentioned in Section Reducing Sugars).

#### 2.7.3. Growth Assessment

The maximum specific growth rate, *µ_max_* (d^−1^), biomass productivity, *P_X_* (cell·mL^−1^.d^−1^), and total substrate consumption (%)were estimated according to the following equations:

(1) $$\mu_{\max}=\frac{\ln X_{2}-{\ln X}_{1}}{t_{2}-t_{1}}$$

where *X*_1_ and *X*_2_ are the number of cell·mL^−1^ at time 1 (*t*_1_) and time 2 (*t*_2_) in the exponential phase, respectively.

(2) $$P_{X}=\frac{X_{2}-X_{1}}{t_{2}-t_{1}}$$

where *X*_1_ and *X*_2_ are the number of cell·mL^−1^ at time 1 (*t*_1_) and time 2 (*t*_2_), respectively.

(3) $$\%\;Substrate\;consumption=\frac{{(C}_{i}-C_{f})\;}{C_{i}}\;\times \;100$$

where *C_i_* and *C_f_* are, respectively, the initial and final concentrations of reducing sugars (g·L^−1^), and *t* represents the time, in days, during which the assay was performed.

### 2.8. Statistical Analysis

Statistical analyses of experimental data were carried out using the GraphPad Prism 9.5.0 software and presented as mean ± standard deviation (SD). One-way analysis of variance (ANOVA), coupled with Tuckey’s post hoc test, was used to determine the statistically significant differences between mean values at a confidence level of 95%. For the comparison of only two conditions, an unpair t-student was performed to determine the statistically significant differences between mean values, also at a confidence level of 95%.

## 3. Results and Discussion

### 3.1. Chemical Characterization of the Food Wastes

To evaluate the possibility of valorizing these FW formulations as nutrient sources for the growth of microalgae, it is crucial to determine their composition, especially regarding the elements that are essential for microalgae growth, such as carbon, nitrogen, phosphorus, potassium, magnesium, and calcium. Food waste is a heterogeneous and complex source of nutrients, having different carbon sources in their composition. However, most microalgae species do not have the capacity to metabolize complex carbon sources, being simple molecules—such as glucose—favored when grown under heterotrophic or mixotrophic conditions. As the present retail FW (Table 1) are, theoretically, rich in different organic compounds, they can be valorized as a direct or indirect source of nutrients (e.g., sugars, nitrogen, phosphorus). Usually, the C:N ratio is a critical nutritional parameter that has to be considered for optimal microalgae growth and accumulation of specific compounds in heterotrophic cultures. Its optimum value varies according to the microalgae species and used culture conditions [25]. According to Table 2, only FW *5* and *6* have C:N ratios lower than 30:1, indicating higher nitrogen levels—as observed by their total percentage. Considering all the food categories in the FW recipes, fish and meat have higher amounts of proteins and, consequently, higher nitrogen percentages [26]. Therefore, these results were somehow expected since FW *5* and *6* have fish in their composition.

Regarding the impact of C:N ratios, Cai et al. [27] demonstrated the effect of different ratios on the growth and protein accumulation of *C. vulgaris* grown under heterotrophic culture conditions using rice hydrolysate. This study showed that nitrogen became a limiting factor for microalgal growth when the C:N ratios exceeded 19:1, resulting in lower biomass productivity. On the contrary, lower ratios were associated with higher protein production since higher nitrogen levels were available for their production. Similarly, Geada et al. [28] also demonstrated that the optimum C:N ratio for the heterotrophic cultivation of *C. vulgaris* 0002 CA (same microalgae strain used in this work) in the commercial medium was 19:1; they also found that a ratio above 50:1 had a significant negative impact on biomass growth. In addition, other studies using different *C. vulgaris* strains showed the impact of this parameter in their growth as well. For instance, Zheng et al. [29] found that the optimal biomass conditions were achieved using a ratio of 25:1, while Singhasuwan et al. [30] reported the best microalgal growth with a C:N ratio of 29:1. Comparing these studies with the results shown in Table 2, most of the FW have values within the range of optimal C:N ratios, for the same species. However, considering that these values were calculated for the total concentration of carbon (regardless of its form), it is hard to compare them directly with the results in the previously reported studies.

In terms of COD, all the FW formulations, except for *3* and *6*, have values higher than 100 g·L^−1^ (Table 2). Nevertheless, all the COD values were under the same range as those reported in the literature for this type of FW. For example, Shin et al. [31] characterized FW-recycling wastewater and obtained COD values between 48.4 and 200.3 g·L^−1^.

In addition to carbon and nitrogen sources, microalgae also need other nutrients for their growth, some of which are presented in Table 3. Generally, one is able to establish an order considering the abundance of each compound in the FW mixtures: K > P > Na > Ca > Mg > Fe.

To understand whether the FW formulations are suitable for use as a nutrient medium for microalgae growth, it is important to compare the concentration of each element quantified with their concentrations on standard culture media typically used. Some of the common media used to grow microalgae species are BG11, BBM, and F/2 [32]. Regarding the compounds and their amount in the different synthetic media, it is possible to calculate the concentration of each element. For example, BG11 medium has in its composition: 0.41 g·L^−1^ of Na, 0.018 g·L^−1^ of K, 0.007 g·L^−1^ of Mg, 0.008 g·L^−1^ of Ca, and 0.007 g·L^−1^ of P [33]; BBM has: 0.21 g·L^−1^ of Na, 0.075 g·L^−1^ of K, 0.004 g·L^−1^ of Mg, 0.003 g·L^−1^ of Ca, 0.0001 g·L^−1^ of Fe, and 0.05 g·L^−1^ of P [34]; F/2 medium has: 0.43 g·L^−1^ of Na, 0.015 g·L^−1^ of K, 0.035 g·L^−1^ of Mg, 0.017 g·L^−1^ of Ca, and 0.001 g·L^−1^ of P [35]. Concerning the concentration of each element in the FW formulations, it is possible to determine the range of values for Ca, K, Mg, Na, and P—0.07–0.31 g·L^−1^, 0.81–2.49 g·L^−1^, 0.03–0.14 g·L^−1^, 0.06–0.79 g·L^−1^, 0.18–0.45 g·L^−1^, respectively. Fe concentrations were low in all the FW formulations. In general, comparing the range of each element in the different standard media and the FW media, we observe that the latter have higher concentrations. Although such concentrations might not be completely bioavailable for microalgae uptake, it is possible to assume that these FW formulations provide a significant part of the required elements for microalgae cultivation in their composition.

**Figure 1:**
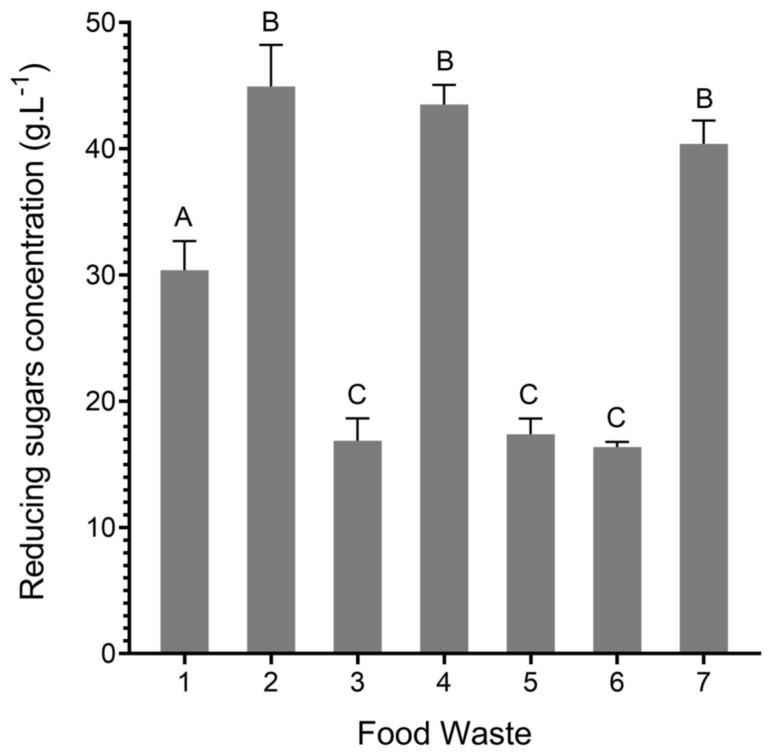
Reducing sugar concentration (g·L^−1^)—determined through DNS methodology—for each untreated FW mixture (1 to 7) solubilized at 250 g·L^−1^. Values are expressed as mean ± standard deviation of three different analyses (*n* = 3). Different letters indicate significant differences between the values (*p* < 0.05).

Analyzing the results of Figure 1, it is possible to verify that FW *2*, *4*, and *7* are not statistically different regarding the concentration of reducing sugars. The same is verified when comparing FW *3*, *5*, and *6*. Interestingly, comparing these results against the overall ratio of each food category in the different FW (Supplementary Materials Figure S1), there is a correlation between the amount of reducing sugars and the portion of fruits, vegetables, dairy, and bakery. For example, FW *2*, *4*, and *7* have higher concentrations of these sugars and similar ratios of the referred food categories.

Curiously, FW *1* also has the same ratios of fruits, vegetables, dairy, and bakery. However, its amount of reducing sugars was statistically different from others. This fact is probably associated with the differences found in the food products utilized within each food category when preparing the FW mixtures (Table 1).

Another essential factor to take into account is the complexity of FW formulations. As mentioned above, the FW formulations are composed of different food products and categories. Therefore, reducing sugars can vary depending on the FW formulations’ composition, meaning that not all reducing sugars might be suitable for microalgae as a carbon source. For instance, it has been proven that simple sugars, such as monosaccharides, have a greater impact on the growth of these microorganisms. Zhang et al. [36] tested the effect of different carbon sources (i.e., monosaccharides, disaccharides, and starch) on the growth and biochemical composition of *Chlorella pyrenoidosa*. The results showed that the growth of this microalgae was enhanced in the presence of glucose, galactose, or fructose, while the use of starch and disaccharides (maltose, sucrose, and lactose) had low or no impact on growth. These findings suggest that microalgae cells have higher efficiency in utilizing simple organic carbon sources. This could be associated with the fact that, globally, simple molecules require less energy for transportation or hydrolysis when compared with more complex ones. For instance, Wang et al. [37] reported that *C. pyrenoidosa* cannot use sucrose as an organic carbon source for its growth or survival under heterotrophic cultivation. Thus, the author tested a co-culture of yeast (*Rhodotorula glutinis*) and microalgae (*C. pyrenoidosa*) to allow the use of sucrose for the heterotrophic cultivation of this microalgae. The yeast hydrolyzed the sucrose into glucose and fructose, which were subsequently used by microalgae cells to grow. Moreover, Freitas et al. [38] tested the potential of pentoses (xylose and arabinose) as carbon sources in the growth and carbohydrate production of different microalgae strains, concluding that these monosaccharides positively affected both. Even though microalgae can use different carbon sources for their growth, glucose is the most effective—enhancing biomass yield—and is usually the main organic carbon source used for heterotrophic or mixotrophic growth of microalgae [39].

### 3.2. Effect of Pretreatments on Microbial Contamination of the FW Formulations

Given that FW formulations have different compounds in their composition, such as organic carbon, that can be used as a source for microbial growth, the possibility of their microbial contamination cannot be overlooked. Therefore, all the FW mixtures were evaluated to detect potential microbial contamination before and after applying different pretreatments (Table 4). It is worth mentioning that this is a qualitative assessment in which the different levels of contamination were selected based on the example illustrated in Figure S2.

All the untreated (WT) samples have shown microbial contamination, where FW *2*, *6*, and *7* exhibited a higher quantity and variety of microorganisms. Based on Table 4 results, the selected pretreatments effectively diminished microbial load. AT was the most effective method among the pretreatments tested since it eliminated contamination in all FW formulations. This process, in addition to temperature, involves pressure (1 bar above the atmospheric pressure). Under these conditions, the microbial cells viability decreased due to the effect of high temperatures on the metabolic activity of cells, leading to their death. In agreement with our results, Carter et al. [40] concluded that the autoclaving process eliminated all microbial contamination in their soil samples, regardless of the type of microorganisms present. Considering the samples treated with COV and the non-treated samples, COV was the least effective treatment when compared with other methods used in our work, as it was only able to eliminate approximately 57% of microbial contamination of the FW formulations. This treatment relies on heat convection and conduction mechanisms to sterilize the FW mixtures [41]. Due to the abovementioned mechanisms, it is possible that the temperature in the sample was not evenly distributed, therefore reducing the treatment’s efficiency. On the other hand, except for FW *2*, OH controlled the contamination in every FW. OH was shown to be a more effective pretreatment when compared with COV, mainly because it provides homogenous heating within a sample without depending on conduction or convection mechanisms [42]. Additionally, the application of electric fields—generated by OH—in microorganisms has proven to be effective in their inactivation. Machado et al. [43] showed that the application of a moderate electric field can affect the transmembrane potential of *E. coli*, leading to changes in the cell structure (e.g., formation of pores and electroporation effects). They claim that this type of phenomenon can be observed if the electric field applied can exceed a threshold of 0.2 to 1 V. They also claimed that exposure to these moderate electric fields has led to profound changes in the biochemical mechanisms of the cells, which could explain cell death. Although Machado and co-workers [43] applied higher electric fields and lower temperatures when compared with those used in the present work (12.5 V.cm^−1^), they proved this technology’s effect on microbial inactivation. Adding the Joule effect creates a synergistic outcome that could promote microbial inactivation, even under low electric fields. This can explain the higher efficiency of OH in controlling microbial contamination compared with the COV process.

Taking into account the aforementioned results, the effectiveness of each treatment can be assigned to its unique characteristics. Consequently, each treatment will subject microorganisms to different stress conditions. For instance, AT involves temperature and pressure conditions, OH combines electric fields and temperature, and COV relies only on temperature.

### 3.3. Effect of Pretreatments on Carbon Source

**Figure 2:**
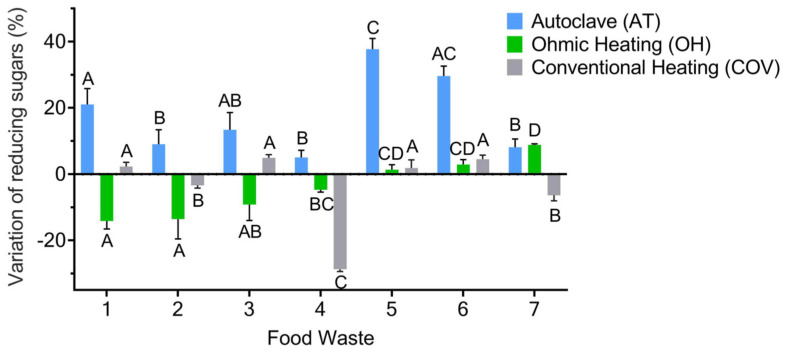
Relative difference in the concentration of reducing sugars (%)—determined through DNS methodology—for every FW formulation after each treatment (AT, OH, and COV), using the concentration of reducing sugars of the non-treated samples as a baseline. Values are expressed as mean ± standard deviation of three different analyses (*n* = 3). Statistical analysis was performed comparing different FW formulations within the same treatment, represented with capital letters. Different letters indicate significant differences between the values (*p* < 0.05).

In addition to the impact of each treatment on the control of microbial contamination, it is also essential to study the effect on the availability of organic carbon sources (reducing sugars). This evaluation was conducted considering the relative difference in the concentration of reducing sugars and taking the concentration of these sugars on the untreated FW samples as a baseline (Figure 2).

According to Figure 2, it is evident that autoclaving had a positive effect on reducing sugar concentration, increasing more than 30% in some FW formulations. Conversely, OH treatment generally decreased the concentration of reducing sugars compared with that obtained in the untreated FW samples. This phenomenon can be explained by the influence of the electric field on certain molecules (e.g., modification of structural conformation of proteins). Some works have proven that OH technology can promote the thermal unfolding of proteins and the rearrangement of their molecular structure. Rodrigues et al. [44] have shown significant changes in milk proteins when subjected to moderate electric fields above 70 °C, causing proteins to unfold. Since the FW formulations subject to OH have proteins in their composition, either derived from dairy products or meat and fish, these could have experienced such unfolding mechanisms and exposed certain protein groups that could react with other macromolecules of the FW formulations. As an example of this molecular interaction, the involvement of proteins and reducing sugars in Maillard reactions has been proven. These reactions are associated with the condensation of reducing sugars with amino groups of proteins, amino acids, or peptides, leading to polymerized proteins and brown pigments (also called melanoidins) [45,46]. The decrease in reducing sugars observed in most of the FW formulations treated with OH can be related to the complexation of these molecules with proteins (Maillard reaction). Nevertheless, the shift of color to brown, usually seen in other products—such as milk—and associated with these reactions was not verified in these FW samples since they are already brown. As for the COV process, globally, it did not have a prevalent influence on reducing sugar concentration compared with the other pretreatments.

Considering the complexity of the FW mixtures, a possible explanation for the higher availability of reducing sugars after autoclaving is due to the hydrolysis effect of high temperatures and pressures on complex molecules (e.g., polysaccharides) usually present in FW. For example, Papadimitriou [47] demonstrated an increase in organic matter solubilization after the autoclave process, possibly associated with the characteristics of this treatment. Moreover, it enhanced the hydrolysis of organic compounds and molecules present in municipal solid waste, corroborating the results obtained in the present work.

**Figure 3:**
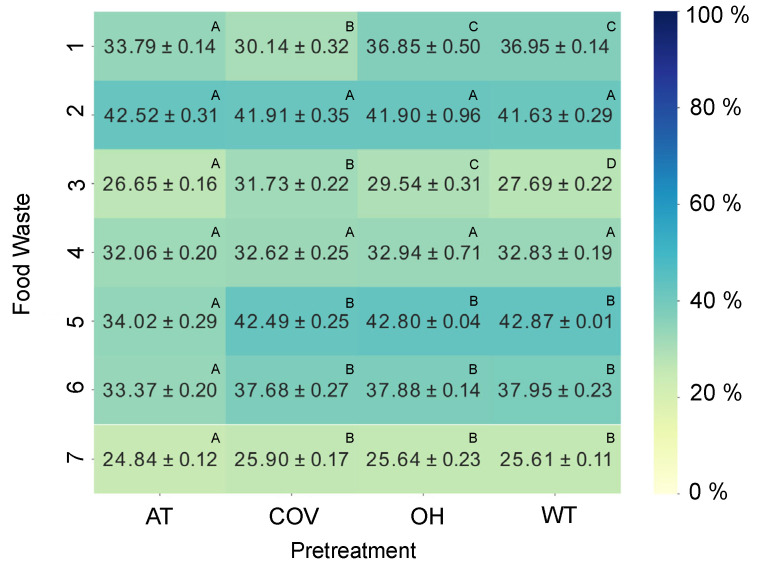
Comparison of the percentage of glucose present in the different FW formulations between pretreatments (autoclaving (AT), conventional heating (COV), ohmic heating (OH), and without treatment (WT)) obtained by HPLC. Values are expressed as mean ± standard deviation of three different analyses (*n* = 3). Statistical analysis was performed comparing different pretreatments within the same by-product, represented with capital letters. Different letters indicate significant differences between the values (*p* < 0.05).

Glucose is the major organic carbon source used for microalgae growth since it is possible to obtain higher biomass yields and productivity with this monosaccharide. Bearing this in mind, a study was conducted to evaluate the percentage of this reduced sugar in the FW formulations and the effect of the different pretreatments on its concentration (Figure 3).

As expected, all the FW formulations have glucose in their composition. This is a positive outcome since its use as the main organic carbon source is favored in the heterotrophic cultivation of microalgae. Analyzing Figure 3, it is possible to verify that FW *2* has the highest glucose amount, which was not influenced by any of the pretreatments applied. On the other hand, FW *3* and *7* had the lowest percentages of glucose. Unfortunately, it was impossible to establish a correlation between the differences in glucose levels/amounts and the quantities of different food categories used in the FW recipes (Figure S1) due to a lack of information provided by the retailer. To confirm that the amount of glucose is associated with the types of foods composing the FW formulations, it would be necessary to have access to the specific quantity of each food product used.

Regarding the effect of the different pretreatments on the availability of glucose, generally, neither COV nor OH contributed to relevant changes in the amount of glucose in the FW formulations. As opposed to this, AT had a negative effect. Considering the results presented in Figure 2, it is possible to assume that the tendencies regarding the effect of the different pretreatments on the concentration of reducing sugars are not due to the variation in the amount of glucose. For instance, after applying the AT process, the concentration of reducing sugars increases in all the FW formulations, as mentioned above. However, this tendency is not seen in the percentage of glucose, which means that the increase in the concentration of reducing sugars seen after the AT process is associated with an increase in other reducing sugars originating from the hydrolysis of organic molecules.

### 3.4. Heterotrophic Growth of C. vulgaris

In order to evaluate the potential of FW formulations on biomass production of *C. vulgaris*, cultivations were performed in 250 mL Erlenmeyer flasks using autoclaved FW *2* and *4* as culture media since these were the ones with the highest concentration of reducing sugars (Figure 1). Additionally, some important growth parameters, such as maximum specific growth rate, maximum cell density, biomass productivity, and substrate (organic carbon) consumption (Table 5), were assessed in order to understand and compare microalgae’s performance using both substrates.

Comparing the growth of *C. vulgaris* 0002 CA in both FW formulations, microalgae cells had higher adaptability to the FW *2* medium since all the growth parameters analyzed were significantly higher. Nevertheless, it is important to note that the lowest growth obtained using FW *4* as a medium did not result from the lack of carbon source since the same concentration of reducing sugars was set for both cases at the beginning of the assay. Also, considering both conditions in terms of total substrate consumption, when using FW *2*, it consumed 8.50 ± 0.01 g·L^−1^ of reducing sugars, while in the case of FW *4*, it only consumed around 3.66 ± 0.38 g·L^−1^. Therefore, the lower growth observed in the second condition is not due to the lack of a carbon source. Regarding previous findings (Figure 3), at least 32% of the reducing sugar concentration corresponds to glucose, meaning that approximately 6.57 ± 0.05 g·L^−1^ of this sugar is available in the FW *4* medium to be used by microalgae cells. Nevertheless, as mentioned above, it only consumed half of the amount of glucose in the medium. Interestingly, the concentration of glucose in FW *2* and the percentage of reducing sugars consumed by *C. vulgaris* using this FW formulation are very similar. This could indicate that the growth of this microalga is associated only with glucose consumption since it seems to have stopped due to the depletion of this sugar in the medium.

Considering both FW recipes (Table 1), a possible explanation for the differences in growth performance may rely upon the specific concentration of each element present in the medium. Therefore, to gain insight, it is important to compare both media in terms of the number of elements (Table 3) and glucose—initial concentration of approximately 8.5 and 6.5 g·L^−1^ for FWs *2* and *4*, respectively. Carbon, nitrogen, and phosphorus are recognized as the primary limiting nutrients in microalgae growth since they are the main nutrients for cellular metabolism and microalgae development. In this sense, it is important to ensure the availability of such elements. A suitable ratio accepted by the scientific community is the so-called Redfield ratio (ratio 106:16:1—molar basis—for C:N:P), which allows a balanced growth of these microorganisms. Additionally, the presence of other elements, even in smaller amounts, can also influence the culture’s behavior. Many of these elements have crucial roles in metabolic pathways for microalgae survival or the production of essential metabolic molecules. For example, P can play an important role in the production of ATP molecules (energy source), and it is used as a building block in the cell membrane of microalgae through phospholipids. On the other hand, K and Mg usually play a similar role as they act as enzyme activators. Additionally, K is involved in the synthesis of proteins and carbohydrates, being an osmotic regulator as well [48,49,50]. This reinforces the importance of a balanced supplementation in the growth of microalgae since the lack or excess of any macro- and micronutrients can suppress the metabolic pathway in which it is involved. However, it is evident that the availability and concentration of these elements are also important factors that influence the growth of microalgae cells.

Comparing both media, it is noticeable that FW *4* has higher concentrations of each element—except iron and nitrogen—and glucose. Therefore, the lower nitrogen concentration boosted the C:N ratio. In general, all the FW formulations had higher ratios than the Redfield ratio, but the value associated with this one (37.22) may have resulted in a more unbalanced supplementation, leading to growth inhibition. For example, the study conducted by Cai et al. [27] focused on the influence of different C:N ratios, ranging from 5:1 to 48:1, on the heterotrophic growth of *C. vulgaris*. It was observed that, at higher ratios, besides inhibiting growth, not all the reducing sugars present in the culture medium were consumed. On the contrary, lower ratios led to a complete consumption of these sugars. Another important aspect of nitrogen relates to its source in the culture medium. Nitrogen can be available in diverse forms, encompassing both inorganic (e.g., ammonium and nitrates) and organic states (e.g., urea). Numerous findings have been reported indicating that the interplay among distinct nitrogen sources can lead to consequential interferences in nitrogen uptake, consequently affecting growth [51]. On the other hand, the possibility of low growth rates as a result of the lack of any of the other elements can be excluded. Alternatively, growth inhibition caused by their excessive concentration can be considered. To understand the veracity of this hypothesis, it is important to compare the FW *4* medium with the standard medium used for the heterotrophic cultivation of this *C. vulgaris* strain [24]. Regarding the concentration of each compound present in the standard medium, it is possible to assess the concentration of each element: 0.08 g·L^−1^ of Ca, 0.22 g·L^−1^ of Fe, 4.84 g·L^−1^ of K, 0.48 g·L^−1^ of Mg, 7.63 g·L^−1^ of Na, and 3.57 g·L^−1^ of P. Analyzing the concentration of each element, it is evident that all the elements are in lower concentrations in FW *4* medium, except calcium. These findings suggest that excess calcium could have a negative effect on the growth of *C. vulgaris*. However, little to no work has been developed on the effect of different calcium concentrations on microalgae growth. Ren et al. [52] concluded that higher concentrations of calcium (higher than 0.098 g·L^−1^) negatively affected Scenedesmus sp. growth under heterotrophic conditions. Even though the microalgae species are different, it is known that calcium is an ion messenger involved in different cell mechanisms, such as cell membrane formation and stability, affecting cell growth and lipid production [53]. Furthermore, according to Geada et al. [28], a study employing experimental designs to optimize all components of a medium applied to industrial production of microalgae revealed a negative effect of Ca element concentration. Particularly, an increase in Ca concentration caused a decrease in the cell concentration of *C. vulgaris*. Considering that calcium concentration in FW *4* is 3.9 times superior, this can indicate that this ion is in excess concentrations. Regarding other elements, the study performed by Geada et al. [28] also revealed favorable outcomes for Mg and Na in the optimization process. It suggests that increasing the concentration of these elements tends to influence biomass concentration positively. Mg and Na play crucial roles in cell development and are associated with physiological processes and metabolic responses. Furthermore, unfavorable concentrations of these elements may result in insufficient C and N consumption. In this sense, the concentrations present in FW *4*, coupled with Ca, can induce a synergistic effect, ultimately leading to lower productivity. Nonetheless, considering the complexity of FW formulation composition, other possible explanations, such as the unavailability of nitrogen or phosphorus sources and the existence of compounds with antimicrobial activity, such as phenols, cannot be overlooked.

## 4. Conclusions

The aim of this research was to utilize FW as a nutrient-rich medium for microalgae growth and assess the impact of different pretreatments on microbial contamination and reducing sugar content. Both the elemental analysis and the concentration of reducing sugars exhibited by the different retail FW demonstrated that these can be used as substrates for the cultivation of microalgae, with glucose concentration ranging between 25 and 43% of the total reducing sugar concentration. Regarding the pretreatments used, the AT process eliminated the contamination in all the FW media while having a positive effect (an increase) on the concentration of reducing sugars. Although promising results were found for the OH process, high microbiological inactivation with shorter exposure times and lower temperatures had a negative impact on the total concentration of reducing sugars (carbon source for microalgae). Maximum cell density—19.9 ± 6.2 × 10^7^ cells·mL^−1^—and biomass productivity—3.22 ± 0.15 × 10^7^ cells·mL^−1^.d^−1^—were obtained by cultivating *C. vulgaris* 0002 CA solely using an FW 2-based medium, without supplementation of any nutrients. Additionally, higher calcium concentrations (≈ 0.31 g·L^−1^) seemed to induce a negative effect on the growth of *C. vulgaris*, causing a decrease of approximately 6.6-fold. Overall, this study has highlighted the potential of applying FW as an alternative culture medium for microalgae growth. However, additional work is needed to assess all the limiting factors possibly interfering with the growth of this microalga when using retail FW as substrate.

## Figures and Tables

**Table 1:** Composition of the different retail FW formulations divided by food category.

FW	Fruits	Vegetables	Dairy	Bakery	Meat	Fish
1	Banana	Lettuce	Liquid yogurt	Bread	-	-
	Apple	Cabbage	Solid yogurt			
	Pear	Turpin greens				
		Broccoli				
2	Strawberry	Tomato	Liquid yogurt	Bread	-	-
	Pear	Lettuce	Solid yogurt			
	Mango	Pepper				
	Banana					
3	Pear	Tomato	Liquid yogurt	Bread	Frozen	-
	Apple	Carrot	Solid yogurt		chicken	
	Pineapple	Other				
	Papaya					
4	Orange	Carrot	Yogurt	Bread	-	-
	Pear	Tomato	Milk			
	Banana	Pumpkin				
	Strawberry					
5	Banana	Pumpkin	Liquid yogurt	Bread	-	Frozen
	Strawberry	Cabbage	Solid yogurt			fish
	Orange					
6	Banana	Pumpkin	Liquid yogurt	Bread	-	Frozen
	Strawberry	Lettuce	Solid yogurt			fish
	Grapefruit	Cabbage				
		Cucumber				
7	Banana	Tomato	Liquid yogurt	Bread	-	-
	Orange	Onion	Solid yogurt			
	Mango	Potato				
		Other				

**Table 2:** Percentage of nitrogen, carbon, and hydrogen, as well as the C:N ratio, of the different retail FW media. COD values are expressed as mean ± standard deviation of three different analyses (*n* = 3).

FW	Nitrogen (%)	Carbon (%)	Hydrogen (%)	C:N Ratio	COD (g·L^−1^) ± SD
1	1.24	38.16	6.56	30.91	101.7 ± 2.9
2	1.25	37.63	7.03	30.23	106.3 ± 19.8
3	1.06	36.28	5.95	34.46	51.3 ± 14.9
4	1.07	39.68	7.00	37.22	102.5 ± 14.2
5	1.51	32.04	5.59	21.25	109.1 ± 3.7
6	1.89	37.18	6.10	19.63	71.6 ± 7.7
7	1.10	35.97	6.24	32.81	106.5 ± 3.4

**Table 3:** Concentration, in mg·mL^−1^, of calcium, iron, potassium, magnesium, sodium, and phosphorus in the retail FW media.

	mg·mL^−1^					
FW	Ca	Fe	K	Mg	Na	P
1	0.20	0.001	1.45	0.06	0.36	0.27
2	0.07	0.001	0.81	0.03	0.06	0.18
3	0.13	0.001	1.51	0.07	0.43	0.35
4	0.31	0.001	2.49	0.14	0.53	0.46
5	0.20	0.001	1.68	0.09	0.73	0.39
6	0.20	0.001	1.72	0.11	0.79	0.45
7	0.19	0.001	2.33	0.11	0.34	0.41

**Table 4:** Effect of autoclave (AT), conventional heating (COV), ohmic heating (OH) and no treatment (WT) on microbiological contamination for the seven FW studied.

FW	WT	AT	COV	OH
1	++	−	++	−
2	+++	−	+	+
3	++	−	−	−
4	++	−	+	−
5	+	−	−	−
6	+++	−	−	−
7	+++	−	++	−

(+) indicates microbial contamination and (−) indicates no microbiological contamination.

**Table 5:** Growth rate (*µ_max_*), maximum cell density, biomass productivity (*P_x_*), and total substrate consumption of *Chlorella vulgaris* 0002 CA cultures grown in the different autoclaved FW media (FW *2* and *4*), with an initial concentration of reducing sugars of 20 g·L^−1^. Values are expressed as mean ± standard deviation of three replicates (*n* = 3). Different letters indicate significant differences between the values of each parameter (row) (*p* < 0.05).

Parameter	FW *2*	FW *4*
*µ_max_* (d^−1^)	0.82 ± 0.02 ^A^	0.22± 0.06 ^B^
Maximum Cell Density (×10^7^ cells·mL^−1^)	19.9 ± 6.2 ^A^	3.02 ± 1.26 ^B^
*P_X_* (×10^7^ cells. mL^−1^·d^−1^)	3.22 ± 0.15 ^A^	0.48 ± 0.29 ^B^
Total substrate consumption (%)	42.45 ± 0.004 ^A^	18.3 ± 1.9 ^B^

## Data Availability

The original contributions presented in the study are included in the article/Supplementary Materials, further inquiries can be directed to the corresponding author.

## Supplementary Materials

The following supporting information can be downloaded at https://www.mdpi.com/article/10.3390/foods13071018/s1, Figure S1: Ratio of the different food categories present in each FW, represented by different colours: (●) fruits; (●) vegetables; (●) dairy products; (●) bakery products; (●) fish; (●) meat; Figure S2: Examples of the different levels of microbial contamination on the FW samples; (+) indicates microbial contamination and (−) indicates no microbiological contamination.
