# Supplementary figures and images for: Comparison of Different Pretreatment Processes Envisaging the Potential Use of Food Waste as Microalgae Substrate
Source: Foods. 2024 Mar 26;13(7):1018. doi: 10.3390/foods13071018 (PMC11011475; doi:10.3390/foods13071018)

FW 1

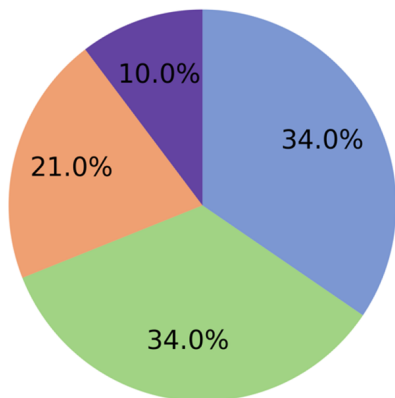

FW 2

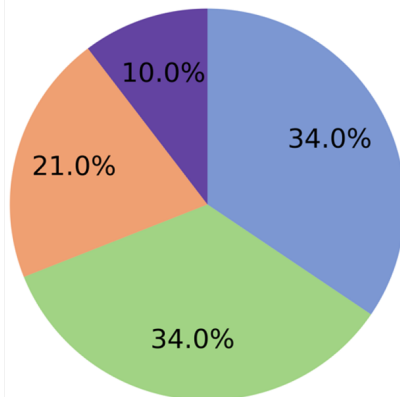

FW 3

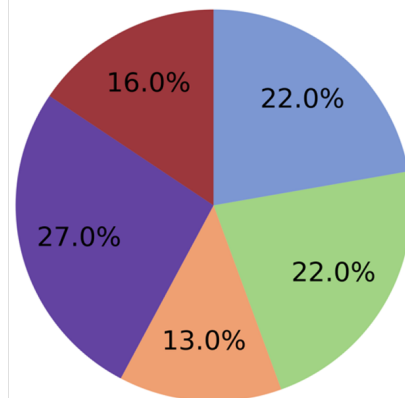

FW 4

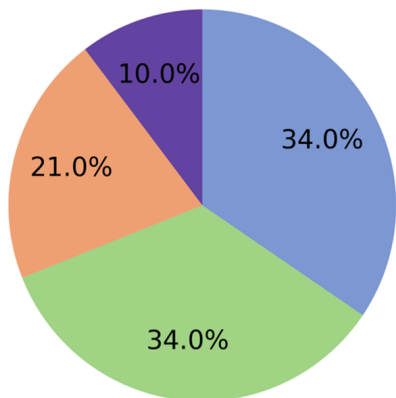

FW 5

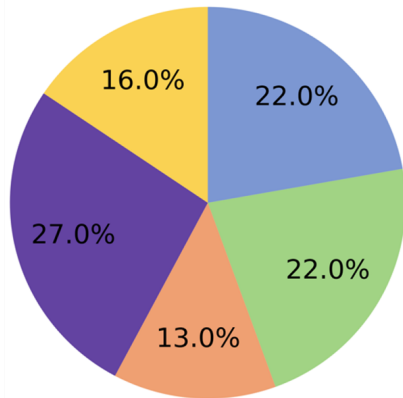

FW 6

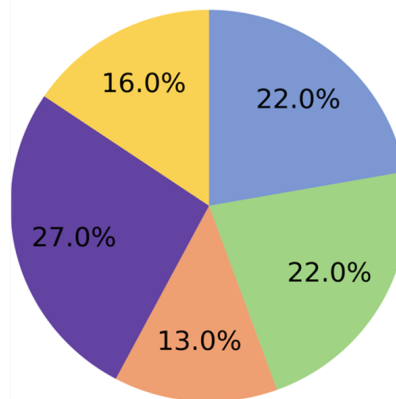

FW 7

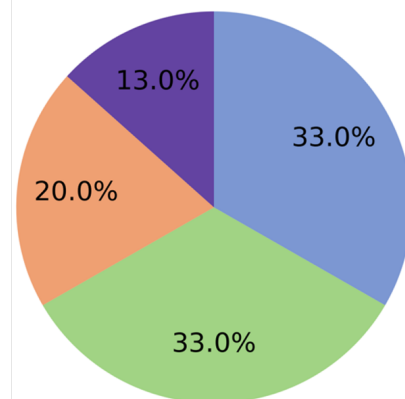

Supplement: Supplementary file 1 [file foods-13-01018-s001.zip › Figure S1 .pdf]

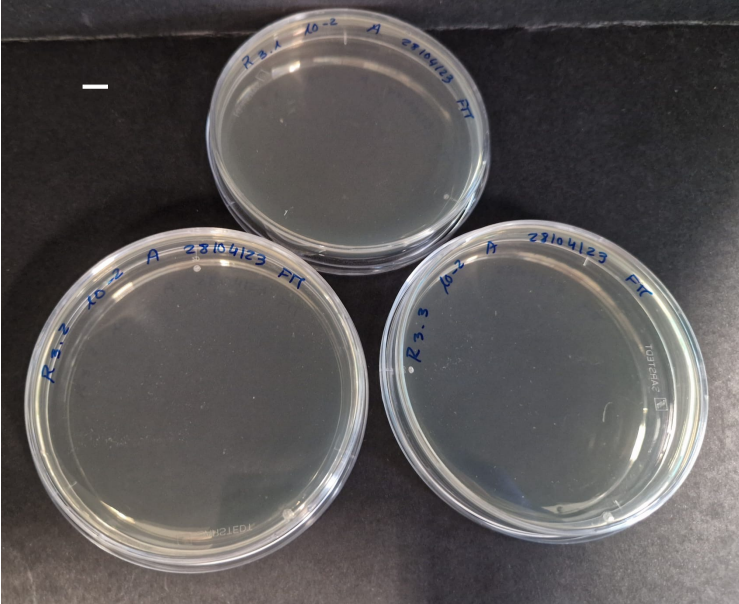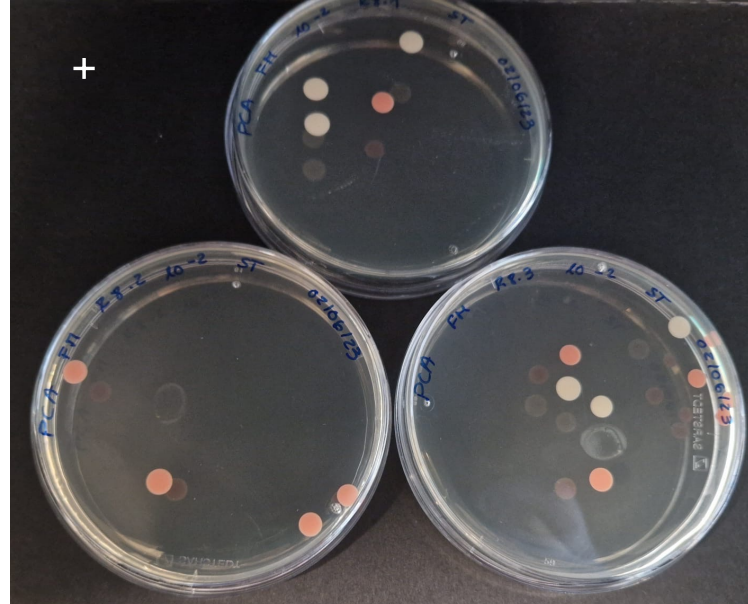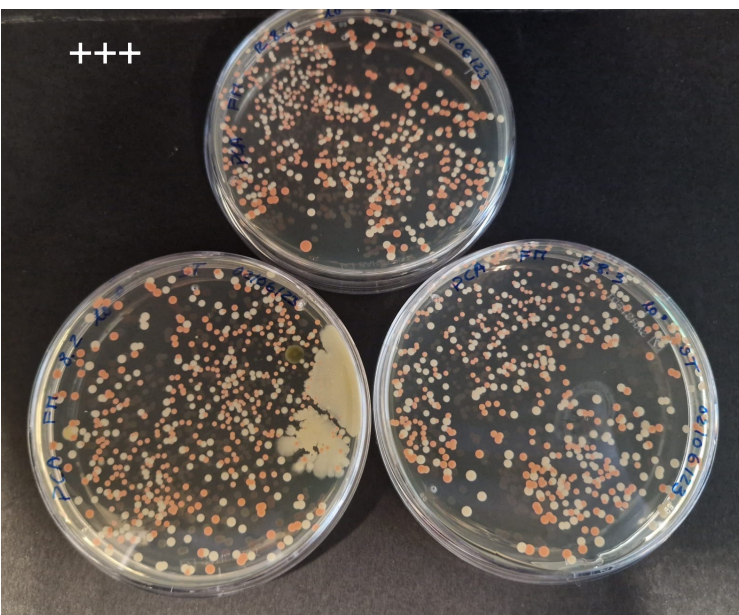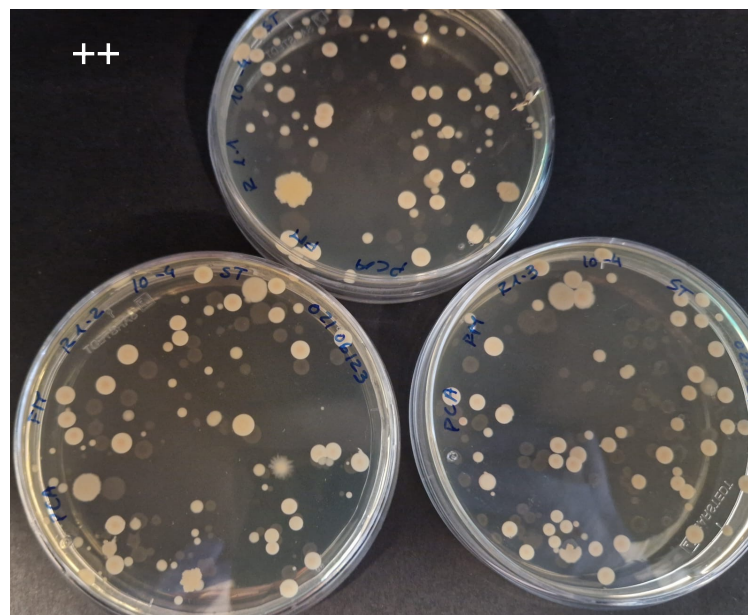

Supplement: Supplementary file 1 [file foods-13-01018-s001.zip › Figure S2.pdf]
